# Mitochondrial superoxide generation induces a parkinsonian phenotype in zebrafish and huntingtin aggregation in human cells

**DOI:** 10.1016/j.freeradbiomed.2018.10.446

**Published:** 2019-01

**Authors:** Brígida R. Pinho, Sara D. Reis, Richard C. Hartley, Michael P. Murphy, Jorge M.A. Oliveira

**Affiliations:** a REQUIMTE/LAQV, Department of Drug Sciences, Pharmacology Lab, Faculty of Pharmacy, University of Porto, 4050-313 Porto, Portugal; b WestCHEM School of Chemistry, University of Glasgow, Glasgow G12 8QQ, UK; c MRC Mitochondrial Biology Unit, University of Cambridge, Cambridge CB2 0XY, UK; d Consortium for Mitochondrial Research (CfMR), University College London, Gower Street, WC1E 6BT London, UK

**Keywords:** Dpf, days post fertilization, HD, Huntington's disease, Htt, Huntingtin, mHtt, mutant huntingtin, MitoPQ, MitoParaquat, NAC, N-acetyl-L-cysteine, OXPHOS, oxidative phosphorylation, PBST, phosphate buffer solution with 0.05% Tween 20, PD, Parkinson's disease, ROS, reactive oxygen species, TH, tyrosine hydroxylase, Mitochondria, MitoParaquat, Zebrafish, Parkinson's disease, Huntington's disease, Superoxide

## Abstract

Superoxide generation by mitochondria respiratory complexes is a major source of reactive oxygen species (ROS) which are capable of initiating redox signaling and oxidative damage. Current understanding of the role of mitochondrial ROS in health and disease has been limited by the lack of experimental strategies to selectively induce mitochondrial superoxide production. The recently-developed mitochondria-targeted redox cycler MitoParaquat (MitoPQ) overcomes this limitation, and has proven effective *in vitro* and in *Drosophila*. Here we present an *in vivo* study of MitoPQ in the vertebrate zebrafish model in the context of Parkinson's disease (PD), and in a human cell model of Huntington's disease (HD). We show that MitoPQ is 100-fold more potent than non-targeted paraquat in both cells and in zebrafish *in vivo*. Treatment with MitoPQ induced a parkinsonian phenotype in zebrafish larvae, with decreased sensorimotor reflexes, spontaneous movement and brain tyrosine hydroxylase (TH) levels, without detectable effects on heart rate or atrioventricular coordination. Motor phenotypes and TH levels were partly rescued with antioxidant or monoaminergic potentiation strategies. In a HD cell model, MitoPQ promoted mutant huntingtin aggregation without increasing cell death, contrasting with the complex I inhibitor rotenone that increased death in cells expressing either wild-type or mutant huntingtin. These results show that MitoPQ is a valuable tool for cellular and *in vivo* studies of the role of mitochondrial superoxide generation in redox biology, and as a trigger or co-stressor to model metabolic and neurodegenerative disease phenotypes.

## Highlights

• MitoPQ is a mitochondria-targeted superoxide generator.
• MitoPQ induces a Parkinsonian phenotype in zebrafish.
• Antioxidant or monoaminergic potentiation strategies rescue MitoPQ induced effects.
• MitoPQ increases huntingtin aggregation in a cell model of Huntington's disease.
• MitoPQ is a useful co-stressor to accelerate disease phenotypes.

## Graphical abstract

**Figure:**
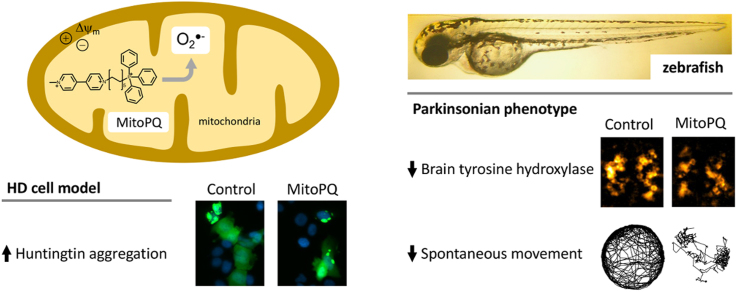

## 1 Introduction

Reactive oxygen species (ROS) were originally characterized as cell-damaging by-products of biological reactions. There is now compelling evidence that ROS also participate in physiological signaling, regulating redox-dependent switches in proteins that govern proliferation, metabolism, gene transcription, inflammation and ageing [11], [20], [46]. Superoxide produced by mitochondrial respiratory complexes is a major source of ROS, involved in signaling to other cellular compartments and in oxidative damage [1]. Available strategies to increase mitochondrial superoxide include respiratory complex inhibitors, which disturb the membrane potential and ATP synthesis [30], and non-targeted redox cyclers, which lead to substantial superoxide production outside mitochondria [6], thereby confounding data interpretation. Thus, the current understanding of the pathophysiological role of mitochondrial superoxide has been limited by the lack of strategies to selectively increase its generation in cells and *in vivo* [20].

MitoParaquat (MitoPQ) is a recently developed molecule comprising the redox cycler paraquat conjugated to a mitochondria-targeting triphenylphosphonium lipophilic cation. MitoPQ accumulates in energized mitochondria and produces superoxide by redox cycling at complex I. It was previously shown *in vitro* that MitoPQ increases mitochondrial superoxide production in cells, and disturbs cardiac function in the isolated perfused mouse heart [41]. There is currently limited information on the *in vivo* effects of MitoPQ, particularly in disease models. *In vivo*, MitoPQ was previously tested only in flies (*Drosophila melanogaster*)*,* where it was found to reduce lifespan, without further functional or behavioral analyses [41]. Here we have performed the first *in vivo* characterization of the effects of MitoPQ in a vertebrate model, the zebrafish (*Danio rerio*). We also assessed the usefulness of MitoPQ in the context of neurodegenerative disease modeling in cells.

Zebrafish is well-suited for *in vivo* drug screening given its advantages, including larval transparency (allowing non-invasive studies of cardiac function), reduced size (facilitating behavioral evaluation), and high genetic and physiological homology to mammals [48]. The zebrafish is increasingly used to study neurodegenerative disorders [35], [50] and associated processes such as mitochondrial dysfunction [2], [36] and oxidative stress [14], [15], [27], [29].

Here we investigate *in vivo* mitochondrial superoxide generation by MitoPQ in zebrafish. Complementing prior evidence that MitoPQ selectively generates superoxide within mitochondria [41], we show here the higher potency of MitoPQ *vs.* paraquat in cells and in zebrafish. We further show that MitoPQ induces a parkinsonian phenotype in zebrafish, with decreased spontaneous movement and brain tyrosine hydroxylase levels. We also tested MitoPQ in a Huntington's disease cellular model where it increased mutant huntingtin (mHtt) aggregation. These results support MitoPQ as a useful tool for investigating superoxide production in pathology in animal and cell models of disease.

## 2 Material and methods

### 2.1 Drugs, solvents and solutions

MitoPQ was synthesised as previously described [41]. Culture media and supplements were from Gibco. Paraquat, N-acetyl-L-cysteine (NAC), desipramine, rotenone, myxothiazol, antimycin A, oligomycin B, and all other drugs or reagents were from Sigma-Aldrich, unless otherwise stated. MitoPQ, rotenone and oligomycin stock solutions were prepared in dimethyl sulfoxide (DMSO), whereas those from desipramine, paraquat, and NAC were prepared in water. Myxothiazol and antimycin stock solutions were prepared in methanol. Experiments in zebrafish larvae were performed with 0.1–0.5% DMSO. Experiments in U2OS cells were performed in presence of 0.1–0.5% DMSO or 0.1% methanol. Control treatment conditions contained the same amount of solvent (DMSO or methanol) as the respective drug treatment conditions.

### 2.2 Zebrafish maintenance and drug treatments

Adult wild-type zebrafish (*Danio rerio*) or larvae were maintained at 28 ± 1 °C with 14 h:10 h light:dark cycles and handled for egg production as we previously described [35], [36]. Fertilized eggs were kept in autoclaved water containing 1 µM methylene blue until 3 days post fertilization (dpf). At 3 dpf, hatched larvae without detectable abnormalities were randomly distributed into multi-well plates, containing autoclaved water and exposed to treatments (MitoPQ, paraquat, NAC, desipramine, or solvent). In all experiments, larvae were imaged daily with a stereomicroscope to assess viability. Dead larvae were removed, and half volume was replaced with freshly prepared treatment solutions [35]. For survival and movement experiments, larvae were exposed to drugs in 24-well plates (5 larvae/500 µL/well), while to assess cardiovascular function, larvae were exposed to drugs in 96-well plates (1 larvae/200 µL/well). In population assays, such as quantification of adenosine nucleotides and dot-blot, as well as for immunofluorescence, larvae were exposed to drugs in 12-well plates (15–30 larvae/1 mL/well).

#### 2.2.1 Cardiovascular function

At 5 and 6 dpf, larvae were anaesthetized with 0.8 M tricaine methanesulfonate and their heartbeats recorded for 30 s, at 10x magnification in a customised Nikon-Prior-Hamamatsu (NPH) Imaging System, composed by an inverted microscope (Eclipse TE300, Nikon), a motorized stage (ProScan, Prior), a CCD camera (ORCA-ER, Hamamatsu), and a monochromator (Polychrome II, Photonics), all controlled by the open source software Micro-Manager (v. 2.0; https://micro-manager.org). Videos were captured at 30 frames per second (fps) and processed with ImageJ (https://imagej.nih.gov/ij/) for assessing atrioventricular coordination and heart rate.

#### 2.2.2 Adenine nucleotides and energy charge

Nucleotide extraction was performed at 5 and 6 dpf, as we previously described [35], but now using a Precellys 24 homogenizer (Bertin Technologies) at 6800 rpm (3 ×20 s, CK28 beads). ATP, ADP and AMP were quantified using HPLC (reversed-phase column; 250 ×4.6 mm Luna 5 µm C18(2) 100 Å; Phenomenex) with a diode-array detector (Agilent 1100 series), measuring 260 nm absorbance. Energy charge was calculated with the formula: ([ATP]+0.5[ADP]) ÷ ([ATP]+[ADP]+[AMP]) [49].

#### 2.2.3 Behavioral evaluation

Measurements of spontaneous swimming and sensorimotor reflexes were performed at 5 dpf, as we previously described [35]. Briefly, individual larvae in 6-well plates were recorded for 10 min at 28 ± 1 °C with a HD digital camera (C525, Logitech). Videos were processed with iWisoft (www.iwisoft.com/videoconverter) and ImageJ to extract time and spatial coordinates. Calculations were automated in customised spreadsheets (Excel, Microsoft) to derive movement parameters: *distance* (cm), *movement speed* (distance ÷ time in movement; mm/s), and *initiations*. After the spontaneous swimming, each larva was tested for reflexes by gently touching with a micropipette tip in the head and in the tail. Immediate swimming after touch was scored as a positive response. Head and tail touches were alternated, spaced by 30 s, and the procedure repeated 10 times for each larva.

#### 2.2.4 Quantification of protein carbonyl groups

Protein extraction was performed at 5 dpf by sonication of 45 larvae in ice-cold lysis buffer (250 mM sucrose; 20 mM HEPES; 3 mM EDTA, pH = 7.5) supplemented with a protease inhibitor cocktail (Fisher Scientific). After 3 freeze-thaw cycles, lysates were centrifuged at 300×*g* (10 min, 4 °C) and carbonyls quantified in the supernatant. Protein was quantified according to Bradford method and carbonyl derivatization was performed as previously described [25], with minor modifications. Briefly, 200 µL of protein extract containing 20 µg protein were incubated with 200 µL of 12% sodium dodecyl sulfate and 400 µL of 20 mM 2,4-dinitrophenylhydrazine hydrochloride (TCI Europe) for 30 min in the dark. After neutralization with 300 µL of 2 M Tris with 18% β-mercaptoethanol, samples containing 1 µg of protein were spotted on a nitrocellulose membrane (Bio-Dot Microfiltration Apparatus, Bio-Rad). Membranes were blocked for 1 h with 5% bovine serum albumin in PBST (phosphate buffer solution with 0.05% Tween 20), and incubated with anti-dinitrophenol antibody (MAB2223, 1:1000, Sigma-Aldrich; 1 h). After washing with PBST, membranes were incubated with secondary antibody (G-21040, 1:4000; Life Technologies; 1 h), and imaged using a Chemiluminescent kit (Novex ECL, Life Technologies) and a ChemiDoc MP (Bio-Rad). Coomassie staining was used for loading control. Densitometric analyses were performed with Image J.

#### 2.2.5 Immunofluorescence

At 5 dpf, larvae were fixed (4% paraformaldehyde, overnight at 4 °C) and processed for immunofluorescence as we previously described [35]. Primary antibodies: anti-acetylated-α-tubulin (T6793, Sigma-Aldrich; 1:1000) or anti-tyrosine hydroxylase (TH) (MAB318, Merck Millipore; 1:500) overnight at 4 °C. Secondary antibody: anti-mouse AlexaFluor-488 (A-11029, Life Technologies) overnight at 4 °C. Larvae mounted in Glycergel medium (DAKO) were imaged in the aforementioned NPH Imaging System. For intensity comparisons, non-saturated images were acquired with identical equipment settings. The mean intensity of immunofluorescence was quantified after background subtraction using ImageJ.

### 2.3 U2OS cell maintenance

Human U2OS cells have been previously used to study mHtt aggregation [4]. These cells are advantageous for their high transfection efficiencies, and also for imaging studies (large, flat cells with clearly-defined nuclear and cytosolic compartments), explaining their widespread use, including in neurodegenerative disease contexts [3], [4], [40]. Here, U2OS cells (ATCC) were cultured in either glucose media (glycolytic conditions) or galactose media (oxidative phosphorylation – OXPHOS – dependent conditions). Glucose media was composed by Dulbecco's Modified Eagle's Medium (DMEM) with low glucose supplemented with 10% foetal bovine serum (FBS) and penicillin/streptomycin. Galactose media was composed by DMEM without glucose supplemented with 10 mM galactose, 2 mM glutamine, 5 mM HEPES, 1 mM sodium pyruvate, 10% FBS, and penicillin/streptomycin. Cells were maintained at 37 °C, in humidified air with 5% CO_2_.

#### 2.3.1 Resazurin metabolism

U2OS cells were seeded at 2.0 × 10^5^ cells/mL in 96-well plates (200 µL/ well). After 24 h, cells were treated with drugs (MitoPQ, paraquat, rotenone, oligomycin, myxothiazol, or antimycin) or solvents (DMSO or methanol). After 20 h of treatment, 40 µM resazurin was added to the cells and its reduction to resorufin assessed by fluorescence readings (Synergy HT, BioTek; 530 nm excitation and 590 nm emission). Readings were performed at 30 min intervals during 4 h (completing 24 h of treatment).

#### 2.3.2 Huntington's disease cell model

U2OS cells in OXPHOS-dependent conditions (galactose media) were seeded at 1.5 × 10^5^ cell/mL in 8-well glass-bottom µ-slides (80827, Ibidi), and transfected as we previously described [17]. For transfection with EGFP-Htt^ex1^Q23 or EGFP-Htt^ex1^Q74 (40261 and 40262, Addgene), cells were treated with Opti-MEM, containing 0.5 µg plasmid DNA, 0.5 µL Lipofectamine LTX and 0.5 µL Plus reagent (Invitrogen) for 45 min (37 °C, 5% CO_2_). 16 h after transfection, cells were treated with 30 µM MitoPQ, 1 µM rotenone or solvent (0.3% DMSO). Nucleus staining with Hoechst 34580 (1 µg/mL, 30 min) and imaging was performed 24 h after treatment. EGFP-Huntingtin (Htt) was excited at 488 nm and Hoechst at 380 nm in the NPH Imaging System. Transfected cells were scored as live or dead through analysis of cell (bright field images) and nuclear (Hoechst staining) morphologies: shrunken cells with condensed DNA were considered dead. Live cells were further divided as having a diffuse or aggregated Htt profile.

#### 2.3.3 Mitochondrial membrane potential and complex analysis

Transfected U2OS cells were equilibrated for 30 min at 37 °C with 50 nM TMRM^+^ (quench mode) in culture media and imaged with the NPH Imaging System. Inhibition of mitochondrial complexes was performed with oligomycin (6 µM; complex V), followed by rotenone (2 µM, complex I), and antimycin A (2 µM, complex III). Uncoupler (CCCP 2 µM) was added at the end of the experiment. EGFP-Htt expressing cells were identified with 488 nm excitation, and time-lapse imaging for changes in TMRM^+^fluorescence was performed at 30 s intervals with 557 nm excitation. Forward ATP synthase operation was detected by increased TMRM^+^ quenching (hyperpolarization) with oligomycin, whereas complex I/III inhibition or CCCP uncoupling was detected by TMRM^+^ dequenching (depolarization) [32], [33].

### 2.4 Statistical analysis

Data are shown as mean ± standard error of the mean (SEM) of the *n* number indicated in Figure legends, except for zebrafish survival data that are shown as Kaplan-Meier representations. Data from zebrafish larvae are from at least 3 different breedings. Concentration-response curves were fitted with non-linear regression. Global differences between two curves were tested with the sum-of-squares F test. For normally distributed data, we used the Student's *t*-test when comparing two groups, and the general linear model with Dunnet's post-Hoc when comparing two or more groups to a control group. For non-normally distributed data, we used the Kruskal-Wallis test with Dunn's post-Hoc for one factor analysis, and the generalized linear model for multi-factorial analyses. Data analyses were performed with Prism 6.0 (GraphPad) or SPSS Statistics version 25 (IBM).

## 3 Results and discussion

### 3.1 MitoPQ activity is mitochondria-dependent and 100-fold more potent than paraquat

**Fig. 1:**
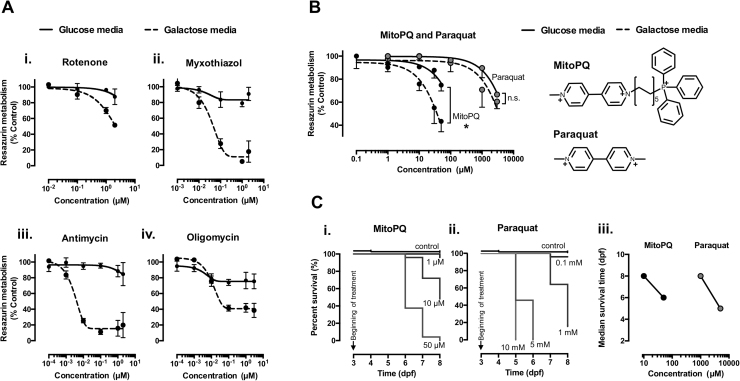
Drug effects on cell metabolism and zebrafish survival. (A) Resazurin metabolism of U2OS cells grown in glucose media (glycolytic conditions) and galactose media (OXPHOS-dependent conditions), after 24 h of treatment with increasing concentration of mitochondrial inhibitors: (*i*) rotenone – complex I inhibitor, (*ii* and *iii*) myxothiazol and antimycin – complex III inhibitors; and (*iv*) oligomycin – ATP synthase inhibitor; *n* = 3–4 independent experiments. (B) Resazurin metabolism of U2OS cells in glucose *versus* galactose media after treatment with MitoPQ and paraquat (left); and their chemical structures (right). Note that the decyl triphenylphosphonium lipophilic cation moiety targets MitoPQ to polarized mitochondria, and that MitoPQ was 100-fold more potent than paraquat in galactose media; *n* = 3 independent experiments, * *P* < 0.05. (C) Kaplan-Meier survival analysis of zebrafish larvae exposed to MitoPQ (*i*), paraquat (*ii*), or solvent (control), *n* = 24–50 larvae; and respective median survival times (*iii*).

MitoPQ was previously shown to induce selective mitochondrial superoxide production in cells [41]. To extend those data, here we tested the mitochondrial-dependency of MitoPQ toxicity in assays with U2OS cells grown in glucose media *versus* galactose media. The galactose media composition (glucose-free) limits glycolysis and increases the cellular dependency on mitochondria (OXPHOS-dependent conditions) [10]. This is evidenced by data in Fig. 1, where mitochondria inhibitors with different targets were all found to induce a much more pronounced inhibition of resazurin metabolism in cells in galactose media (Fig. 1A*i-iv*). In agreement with a mitochondria-dependent activity, MitoPQ was significantly more potent in OXPHOS-dependent than in glycolytic conditions (Fig. 1B). In contrast, non-targeted paraquat showed no significant difference in potency between conditions (Fig. 1B). Moreover, MitoPQ was 100-fold more potent than paraquat: 10 μM MitoPQ induced comparable effects to 1 mM paraquat (Fig. 1B). This difference in potency is consistent with previous data comparing the hydrogen peroxide production induced by MitoPQ *vs.* paraquat from isolated heart mitochondria [41].

**Fig. S1:**
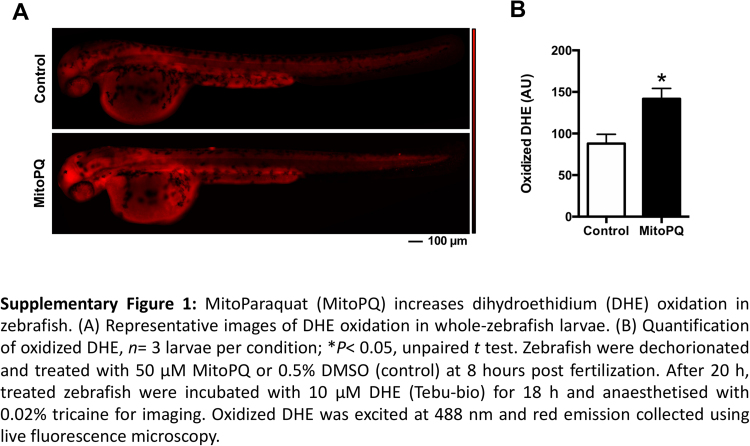
.

We next performed *in vivo* testing of MitoPQ in a vertebrate model. MitoPQ-treated zebrafish presented increased hydroethidine (dihydroethidium; DHE) oxidation, indicating increased intracellular oxidant formation [55] (Supplementary Fig. 1). Kaplan-Meier analysis of zebrafish larvae continuously exposed to MitoPQ or paraquat from 3 to 8 dpf revealed that both compounds induced time- and concentration-dependent effects on survival (Fig. 1C*i,ii*). In agreement with the data from U2OS cells, MitoPQ was 100-fold more potent that paraquat in zebrafish (Fig. 1C*iii*). Taken together, these results are consistent with the mitochondrial accumulation of MitoPQ driven by its lipophilic cation moiety (Fig. 1B; [41]), explaining both its mitochondrial-dependent effects and its higher potency than paraquat.

### 3.2 MitoPQ induces a parkinsonian phenotype in zebrafish

**Fig. 2:**
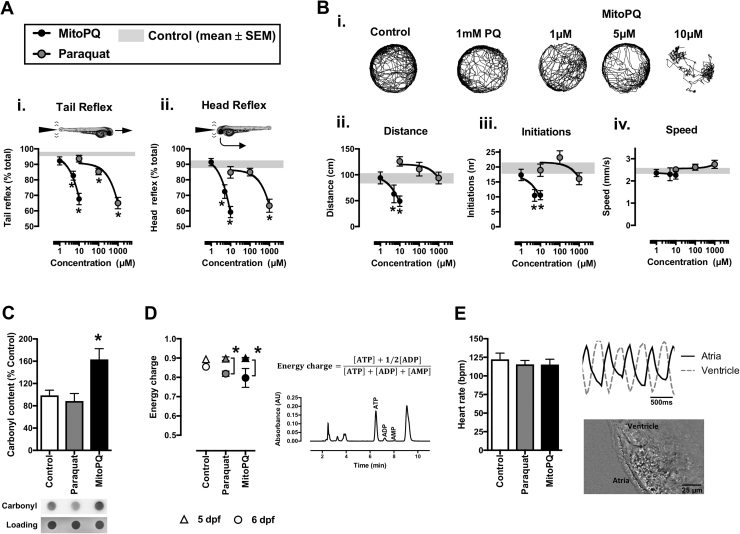
Behavioral, functional and biochemical parameters in zebrafish treated with MitoPQ *vs.* Paraquat. Zebrafish were treated with the indicated concentrations of MitoPQ and paraquat, or solvent (control), from 3 dpf until measurements (at 5 or 6 dpf). (A, B) Sensorimotor reflexes and spontaneous movement at 5 dpf: (A*i*) tail and (A*ii*) head reflexes; (B*i*) representative trajectories; (B*ii*) distance; (B*iii*) initiations, and (B*iv*) movement speed; *n* = 27–78 larvae; *P < 0.05 *vs.* control. (C) Protein carbonyl groups measured by dot-blot at 5 dpf. *n* = 3 independent experiments, *P < 0.05 *vs*. control. (D) Left – energy charge values, calculated with the indicated formula, at 5 dpf (triangles) and 6 dpf (circles); Right – representative HPLC chromatogram with adenine nucleotides; *n* = 3–4 independent experiments. (E) Heart rate and atrioventricular coordination at 6 dpf; n = 16–19 larvae.

To characterize the *in vivo* phenotypes induced by MitoPQ in zebrafish, we first monitored its effects upon sensorimotor reflexes and spontaneous swimming. Larvae treated for 48 h (3–5 dpf) with MitoPQ (1–10 µM) or paraquat (10–1000 µM) showed a concentration-dependent reduction in escape responses evoked by either tail- or head-touch stimulation (Fig. 2A). These results indicate an impairment in evoked sensorimotor reflexes for both compounds, with 10 μM MitoPQ again presenting similar effects to 1 mM paraquat. Interestingly, when comparing effects on spontaneous movement, MitoPQ but not paraquat reduced the travelled distance and the number of initiations of movement, without altering movement speed (Fig. 2B). To further investigate the *in vivo* effects of MitoPQ we studied additional biochemical and functional parameters, comparing 10 μM MitoPQ with 1 mM paraquat.

Treatment with MitoPQ (3–5 dpf) induced a significantly higher protein carbonyl content in zebrafish larvae than paraquat (Fig. 2C), which indicates a higher level of oxidative damage that contributes to the reduced spontaneous movement with MitoPQ, but not paraquat (Fig. 2B). To investigate if differences in energy availability might explain the reduced movement with MitoPQ we measured adenine nucleotide levels and calculated the energy charge. Treatment with either MitoPQ or paraquat from 3 to 5 dpf evoked no detectable changes in energy charge, although extending treatment to 6 dpf did significantly decrease the energy charge (Fig. 2D). This is a likely consequence of cumulative toxicity, together with the time-dependent depletion of yolk reserves that facilitates detection of drug-induced metabolic dysfunction.

We next tested if treatment with MitoPQ or paraquat affected zebrafish heart function, but neither treatment changed heart rate nor atrioventricular coordination, even when extended to 6 dpf (Fig. 2E). This lack of effect upon the zebrafish heart rate is compatible with previous data from the isolated mouse heart, where direct exposure to MitoPQ did not change the heart rate, in spite of disturbing diastolic pressures and coronary flow [41]. Here we cannot exclude the possibility that MitoPQ disturbs pressure and flow of the zebrafish heart (which has a single atria and ventricle), but our imaging method allows detection of changes in atrioventricular coordination induced by mitochondrial inhibitors [36] and such changes were not found in the MitoPQ-treated zebrafish. Thus, the reduced movement phenotype in MitoPQ-treated larvae at 5 dpf is unlikely to result from a widespread collapse of energy availability (preserved energy charge in whole-organism measurements) nor from generalized muscular impairment (preserved heart rate and coordination). Nevertheless, there could still be major metabolic changes taking place in vulnerable tissues, which prompted us to investigate if MitoPQ was compromising neuronal activity.

**Fig. 3:**
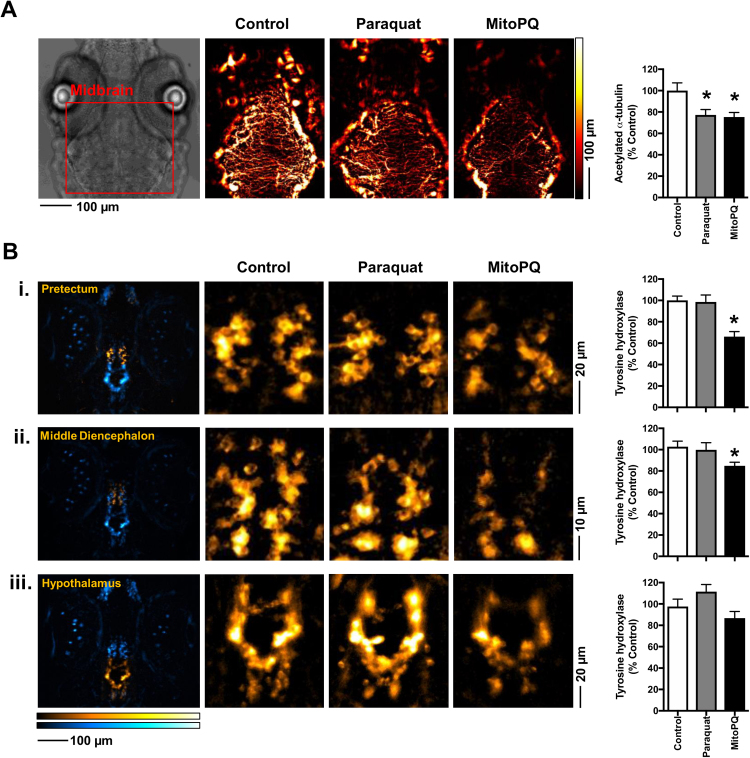
Brain levels of acetylated-α-tubulin and tyrosine hydroxylase (TH) in zebrafish treated with MitoPQ *vs.* paraquat. Larvae were treated from 3 to 5 dpf with 10 µM MitoPQ, 1 mM paraquat, or solvent (control). Acetylated-α-tubulin and TH were quantified by immunofluorescence at 5 dpf. (A) Representative images and quantification of acetylated-α-tubulin in the midbrain; *n* = 7–11 larvae, *P < 0.05 *vs.* control. (B) Representative images of TH-positive neurons and quantification of TH levels in (i) the pretectum, (ii) middle diencephalon and (iii) hypothalamus. The first column shows global TH staining in blue, with a yellow highlight for the region under analysis; *n* = 16–18 larvae, *P < 0.05 *vs*. control.

Using tubulin acetylation levels as an indicator of neuronal structure and function, we found significant decreases in labelling in the midbrain region with either MitoPQ or paraquat treatment, (Fig. 3A), which is compatible with both compounds impairing sensorimotor reflexes. Using tyrosine hydroxylase (TH) levels as a marker for dopaminergic neuronal clusters [43], we found that MitoPQ, but not paraquat, significantly decreased brain TH levels, particularly in the pretectum and middle diencephalon (Fig. 3B). These two neuronal clusters express the dopamine transporter [21] and are the most affected in zebrafish exposed to the dopaminergic toxin MPP^+^ [43]. Thus, the differential impact of MitoPQ and paraquat upon these neuronal clusters could explain their differential effects upon spontaneous movement.

These results show that MitoPQ induces *in vivo* oxidative damage (increased protein carbonyl content) and a parkinsonian phenotype in zebrafish, particularly the reduced mobility and decreased brain levels of tyrosine hydroxylase that are common features of Parkinson's Disease (PD) [22]. These phenotypes suggest that the mitochondrial superoxide production induced by MitoPQ impairs dopaminergic neurotransmission. This is in agreement with dopaminergic neurons being highly vulnerable to oxidative stress [52], [54], and with PD patients presenting oxidative stress markers and monoaminergic dysregulation [13], [28], [31].

### 3.3 Rescue of MitoPQ-induced phenotypes with antioxidant or monoaminergic potentiation strategies

**Fig. S2:**
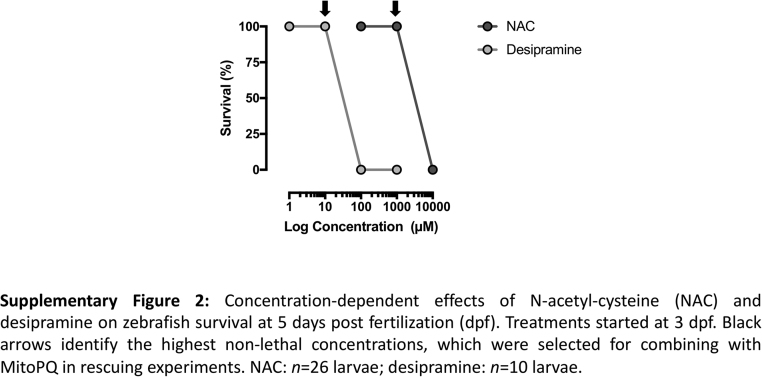
.

Given the pro-oxidant properties of MitoPQ and the sensorimotor phenotypes associated with reduced TH (a key enzyme in the synthesis of dopamine and other monoamine neurotransmitters), we tested if these phenotypes could be rescued with antioxidant or monoaminergic potentiation strategies. For this purpose, we used the antioxidant N-acetyl-L-cystein (NAC; [44]) and desipramine – an inhibitor of the reuptake of monoamine neurotransmitters [9]. We selected the concentrations of 1 mM NAC and 10 µM desipramine based on previous determination of efficacy [26], [34], [42], [44], and lack of effect on zebrafish survival under our experimental conditions (Supplementary Fig. 2).

**Fig. 4:**
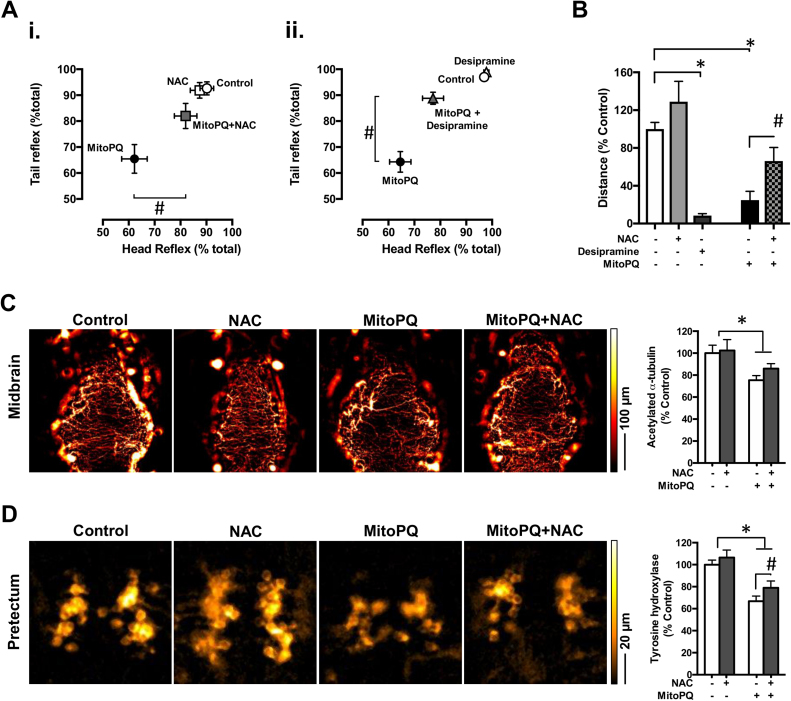
Effects of N-acetyl-L-cystein and desipramine on the behavioral and neurochemical phenotypes of MitoPQ-treated larvae. Larvae were treated from 3 to 5 dpf with solvent (control), 10 µM MitoPQ, 1 mM N-acetyl-L-cystein (NAC), or 10 µM desipramine, alone or in the indicated combinations. (A) Sensorimotor reflexes at 5 dpf; (B) Spontaneous swimming distances at 5 dpf; *n* = 19–54 larvae. (C) Representative acetylated-α-tubulin immunofluorescence in the midbrain and its quantification; *n* = 7–11 larvae. (D) Representative tyrosine hydroxylase immunofluorescence showing neuronal clusters in the pretectum and associated quantifications; *n* = 13–18 larvae. *P < 0.05 *vs.* control; ^#^*P* < 0.05 *vs.* MitoPQ.

NAC or desipramine alone, from 3 to 5 dpf, had no effect on zebrafish sensorimotor reflexes, but in co-treatment with MitoPQ they significantly rescued the impairment of head or tail touch responses by MitoPQ (Fig. 4A). NAC alone evoked no changes in spontaneous movement, whereas desipramine alone significantly decreased the swimming distance (Fig. 4B). We therefore focused on co-treatment with NAC and found that it significantly attenuated the spontaneous movement impairment evoked by MitoPQ (Fig. 4B). We next tested if this movement recovery correlated with neurochemical changes. Although NAC co-treatment failed to significantly recover alpha-tubulin acetylation in the midbrain (Fig. 4C), it significantly increased TH levels in the pretectum (Fig. 4D) – the region showing the largest decrease evoked by MitoPQ (Fig. 3B). Mechanistically, the superoxide generated by MitoPQ is rapidly converted to hydrogen peroxide, which crosses mitochondrial membranes and generates other reactive species that induce widespread oxidative damage [30], [46], [53]. NAC may quench this widespread damage by being a source of sulfhydryl groups that scavenge free radicals and protect protein disulfide bonds [12], [44].

These results show that the Parkinson's like phenotypes induced by MitoPQ in zebrafish can be partially ameliorated by the indicated pharmacological strategies (antioxidant and monoaminergic potentiation), highlighting the potential of MitoPQ as a tool for *in vivo* redox biology research. We next decided to test MitoPQ in another experimental model of disease.

### 3.4 MitoPQ increases huntingtin aggregation in a cell model of Huntington's disease

Given that treatment with MitoPQ generated a parkinsonian phenotype in zebrafish, which may also be interpreted as accelerated ageing [7], we decided to test how treatment with MitoPQ affects survival of a cell model of Huntington's disease (HD) and the proteostasis of the disease associated protein – huntingtin (Htt). As with PD, HD pathophysiology includes mitochondrial dysfunction [18] and age-dependent neurodegeneration [24]. HD progresses with increased formation of mutant Htt aggregates that evoke a slow, necrotic death [39].

**Fig. 5:**
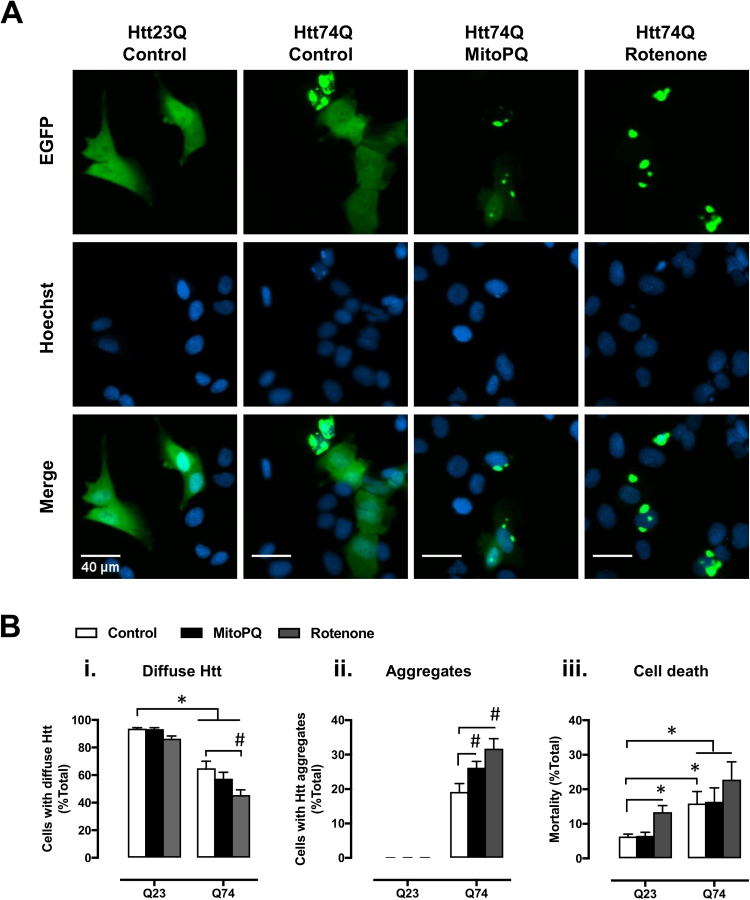
Effects of MitoPQ on huntingtin proteostasis and cell viability in a HD cell model. U2OS cells transfected with GFP-Htt^ex1^Q23 or GFP-Htt^ex1^Q74 were treated for 24 h with 30 µM MitoPQ, 1 µM rotenone, or solvent (control). (A) Huntingtin expression (EGFP) and nucleus staining (Hoechst 34580). Representative images show cells with Htt in diffuse form (Q23 and Q74) and Htt aggregates (observed only with Q74). (B) Quantification of live cells presenting (*i*) only diffuse huntingtin or (*ii*) containing aggregates; and (*iii*) cell death. *n* = 6 independent experiments, * *P* < 0.05 *vs.* Q23; ^#^*P* < 0.05 *vs.* Q74.

To model HD, we used U2OS cells expressing GFP-tagged wild-type (Q23) or mutant (Q74) Htt constructs, in OXPHOS-dependent conditions (galactose media), and treated with MitoPQ for 24 h. Solvent alone and rotenone were used for comparison with the effects of MitoPQ. Concentrations of 30 µM MitoPQ and 1 µM rotenone produced similar effects on metabolism in OXPHOS-dependent conditions (Fig. 1A *vs.* C). Under control conditions, cells expressing Q23-Htt showed a diffuse GFP distribution with no detectable aggregates (Fig. 5A). In contrast, cells expressing Q74-Htt showed aggregates and a significantly higher cell death than Q23-Htt cells (Fig. 5B). Treatment with MitoPQ increased Q74-Htt aggregation without increasing cell death in cells expressing Q23- or Q74-Htt. This differs from treatment with rotenone where we observed a significant increase in cell death, particularly in cells expressing Q23-Htt.

**Fig. 6:**
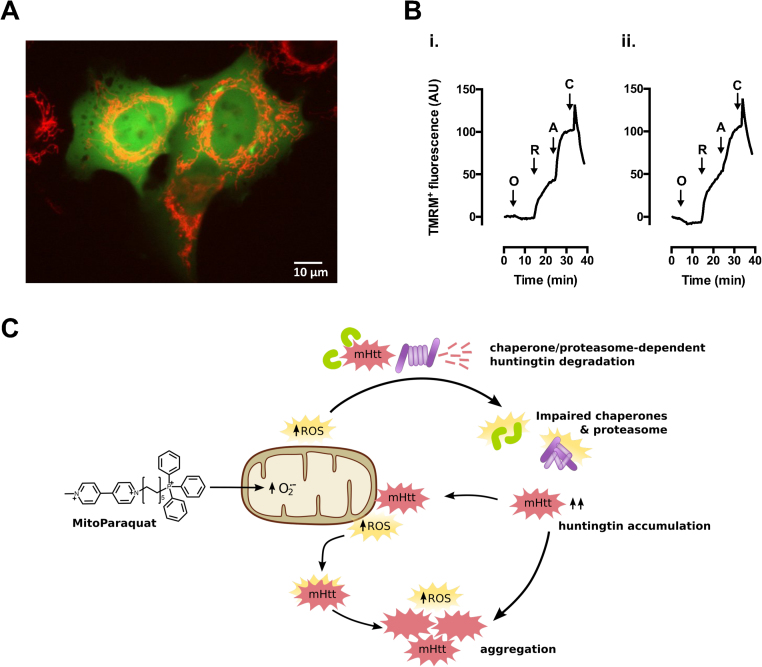
Mitochondria and ROS-mediated impairment of huntingtin proteostasis. (A) Representative U2OS cells expressing diffuse (left) and aggregated (right) mHtt-EGFP (green) in proximity of TMRM^+^-stained mitochondria (red). (B) Representative tracings of changes in TMRM^+^ fluorescence of U2OS cells exposed to the indicated drugs: O, 6 µM oligomycin; R, 2 µM rotenone; A, 2 µM antimycin A; C, 2 µM CCCP; (i) non-transfected cell; (ii) cell expressing mHtt-EGFP, 24 h-post transfection; tracings are representative of at least 10 cells per condition. (C) Mitoparaquat promotes mitochondria superoxide formation [41], arguably accelerating a process that is also thought to occur in the presence of mutant huntingtin (mHtt; [38], [51]). Mitochondria-derived reactive-oxygen species (ROS) impair the ubiquitin-proteasome system [16], [45], [8], and may also impair chaperones [23], [47], limiting mHtt degradation and thereby promoting its accumulation and aggregation. Increased levels of mHtt promote its interaction with mitochondria, and ROS formation [38], [51]. Such ROS, together with those generated from mHtt aggregation [19] may modify mHtt directly, further promoting its aggregation [23]. (For interpretation of the references to color in this figure legend, the reader is referred to the web version of this article).

How might MitoPQ promote mHtt aggregation? While H_2_O_2_ diffusion from MitoPQ-challenged mitochondria might modify mHtt directly, promoting its aggregation [23], particularly near mitochondria (Fig. 6A), there is currently more evidence for indirect effects of ROS *via* impairments in the proteostasis machinery (Fig. 6C). Oxidative stress was found to promote mHtt aggregation in association with proteasomal dysfunction [16] and, particularly, mitochondria-derived ROS was found to reduce the activity of the ubiquitin-proteasome system [45], [8]. Additionally, mitochondria-derived ROS might promote mHtt aggregation by compromising the activity of molecular chaperones, which are vulnerable to ROS-mediated oxidation [47].

These results indicate that the use of MitoPQ as a co-stressor, to accelerate disease phenotypes, has advantages over classical mitochondrial inhibitors such as rotenone, namely by avoiding cell death under control conditions. Further, these results suggest that MitoPQ is an interesting tool to study the effects of increased mitochondrial superoxide production on huntingtin proteostasis, and possibly that of other mutant proteins in disease models.

It is important to note that neurodegenerative disorders are associated with decreased mitochondrial membrane potential [5]. Since MitoPQ uptake into mitochondria depends on mitochondrial membrane potential, it may be more useful in models of early stage disease where mitochondrial membrane potential is still maintained at normal levels. In our experimental conditions, at the time of treating with MitoPQ, cells expressing either diffuse or aggregated mHtt retained mitochondrial membrane potential (TMRM^+^ staining) and were net ATP generators (oligomycin hyperpolarization) (Fig. 6B). As with other drugs or probes influenced by mitochondrial membrane potential [37], the mitochondrial polarization status should thus be taken into consideration when using MitoPQ.

## 4 Conclusions

The generation of mitochondrial superoxide plays an important role in ROS signaling and oxidative damage, but the lack of strategies for its selective generation have limited advancement in this field of research. The mitochondrial superoxide generator MitoPQ was recently developed as a tool to overcome this limitation, and here we present its first *in vivo* study in a vertebrate model. MitoPQ induces a parkinsonian phenotype in zebrafish larvae, supporting a contribution of mitochondrial superoxide generation to the development of PD. MitoPQ also led to an increase in mutant huntingtin aggregation in a cell model. These data suggest that MitoPQ can accelerate ageing-associated processes *in vivo* and in cell models. MitoPQ is thus an interesting co-stressor to accelerate disease phenotypes, and a valuable tool to investigate the role of mitochondrial superoxide generation in physiological and pathological processes, using cellular and *in vivo* models.
